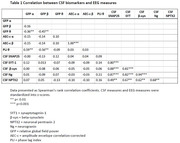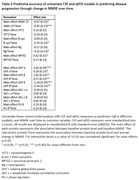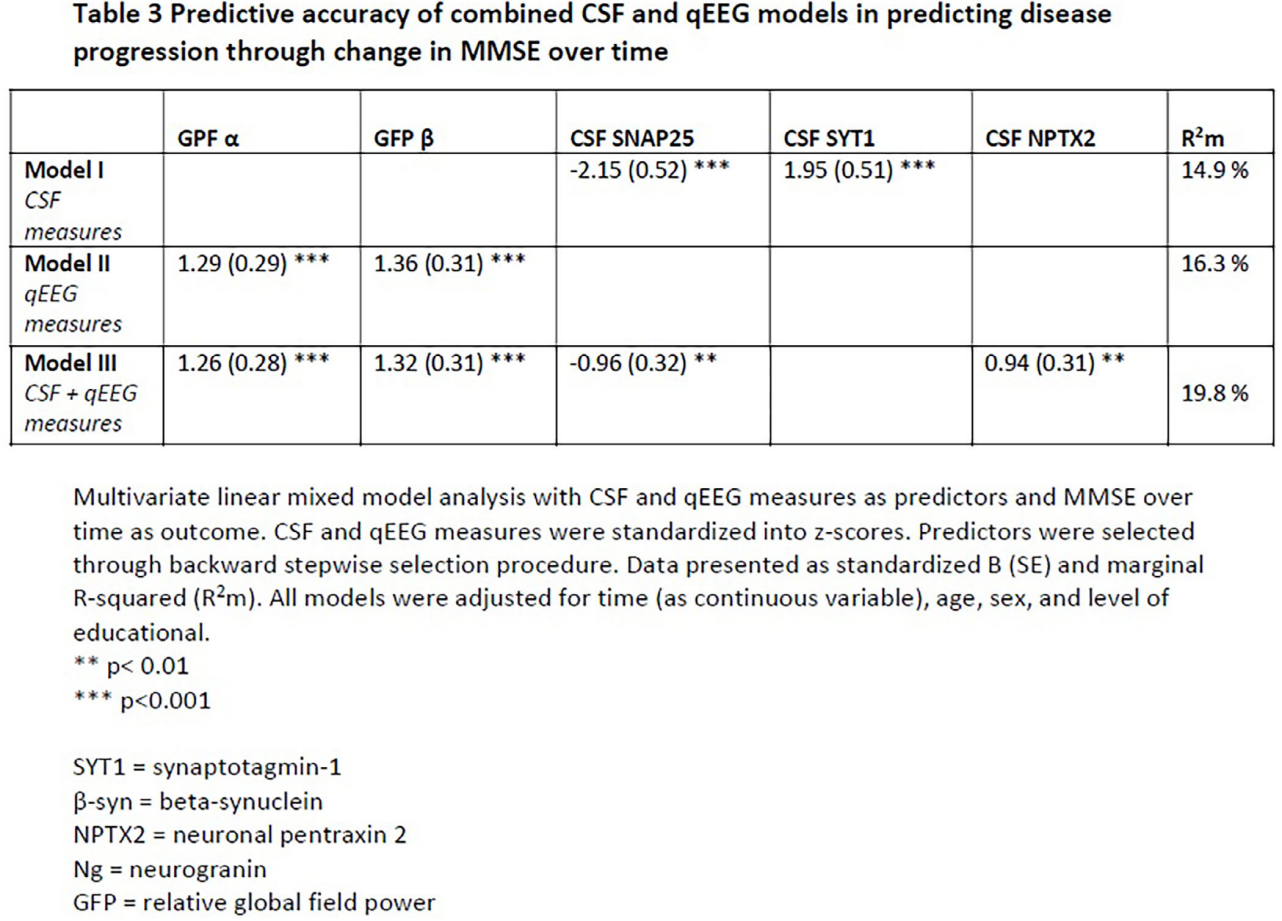# Combination of CSF synaptic biomarkers and qEEG leads to a better prediction of disease progression in Alzheimer’s disease

**DOI:** 10.1002/alz.088895

**Published:** 2025-01-09

**Authors:** Vidhusan Kandiah, Alida A Gouw, Johanna Nilsson, Charlotte Teunissen, Jos W.R. Twisk, Kaj Blennow, Henrik Zetterberg, Wiesje M. van der Flier, Ann Brinkmalm, Flora H. Duits

**Affiliations:** ^1^ Amsterdam UMC, location VUmc, Amsterdam Netherlands; ^2^ Amsterdam UMC location Vrije Universiteit Amsterdam, Department of Clinical Neurophysiology and MEG Center, Neurology, De Boelelaan 1117, Amsterdam Netherlands; ^3^ Amsterdam UMC location Vrije Universiteit Amsterdam, Alzheimer Center Amsterdam, Neurology, De Boelelaan 1117, Amsterdam Netherlands; ^4^ Amsterdam Neuroscience, Neurodegeneration, Amsterdam Netherlands; ^5^ Clinical Neurochemistry Laboratory, Sahlgrenska University Hospital, Mölndal, Sweden, Mölndal Sweden; ^6^ Neurochemistry Laboratory, Department of Laboratory Medicine, Vrije Universiteit Amsterdam, Amsterdam UMC, Amsterdam, North Holland Netherlands; ^7^ Alzheimer Center Amsterdam, Neurology, Vrije Universiteit Amsterdam, Amsterdam UMC, Amsterdam Netherlands; ^8^ Amsterdam UMC, location VUmc, Department of Epidemiology and Data Science, Amsterdam Netherlands; ^9^ Institute of Neuroscience and Physiology Sahlgrenska Academy at the University of Gothenburg, Gothenburg Sweden; ^10^ Clinical Neurochemistry Laboratory, Sahlgrenska University Hospital, Mölndal Sweden; ^11^ Wisconsin Alzheimer’s Disease Research Center, University of Wisconsin School of Medicine and Public Health, Madison, WI USA; ^12^ Institute of Neuroscience and Physiology, Sahlgrenska Academy at the University of Gothenburg, Mölndal, Gothenburg Sweden; ^13^ UCL Institute of Neurology Queen Square London UK, London United Kingdom; ^14^ Hong Kong Center for Neurodegenerative Diseases, Hong Kong China; ^15^ Amsterdam Neuroscience, Vrije Universiteit Amsterdam, Amsterdam UMC, Amsterdam Netherlands; ^16^ Department of Epidemiology and Biostatistics, Amsterdam UMC, Amsterdam Netherlands; ^17^ Department of Psychiatry and Neurochemistry, Institute of Neuroscience and Physiology, The Sahlgrenska Academy at the University of Gothenburg, Gothenburg Sweden

## Abstract

**Background:**

Synaptic dysfunction plays an important role in Alzheimer’s disease (AD) and cognitive decline. We investigated the association between cerebrospinal fluid (CSF) synaptic protein levels and quantitative EEG (qEEG) measures, two modalities to measure synaptic dysfunction in AD pathology. We assessed combined and independent prognostic value of both modalities for cognitive decline along the AD continuum.

**Method:**

We included 114 subjects with SCD or MCI (n=38 with normal Aβ42 [A‐], n=38 with abnormal CSF Aβ42 [A+], and 38 patients with AD dementia) from the Amsterdam dementia cohort, with available CSF and EEG at baseline, and more than one year clinical follow‐up. We assessed associations between CSF synaptic proteins (neurogranin, β‐synuclein, neuronal pentraxin 2 [NPTX2], synaptotagmin‐1 [SYT1], SNAP25) and qEEG measures (Global field power [GFP] ‐α,‐β,‐θ; Amplitude envelope correlation‐corrected [AEC‐c] α, AEC‐c β, and phase lag index [PLI] θ), all standardized into z‐scores, using Spearman correlations. We performed linear mixed models to assess univariate and multivariate associations with cognitive decline (i.e. repeated MMSE) along the AD continuum. We used backwards stepwise selection in multivariate models and calculated marginal R‐squared to compare prognostic accuracy.

**Result:**

There were no correlations between CSF and qEEG markers (Table 1). In univariate models SNAP25, beta‐synuclein, neurogranin, GFP alpha, beta and theta emerged as predictors of cognitive decline (Table 2). SNAP25 showed the largest effect (stB[SE]=‐0.45 [0.13], p<0.001). For GFP‐alpha and –beta effects were reversed (0.29[0.13], p<0.05 and 0.37[0.13], p<0.01 respectively). Multivariate models combining CSF and qEEG measures improved prognostic accuracy compared to CSF or EEG only models (Table 3; R^2^m = 19.8%, StB[SE] = 1.26[0.28] for GFP‐α, StB[SE] = 1.32[0.32] for GFP‐β, StB[SE] = ‐0.96[0.32] for SNAP25 and StB[SE] = 0.94[0.31] for NPTX2, all p<0.01).

**Conclusion:**

CSF synaptic proteins and qEEG measures were not directly correlated to each other, but both modalities were associated to cognitive decline. This suggests they represent different aspects of synaptic pathology in AD. The combination of both modalities showed increased prognostic accuracy over CSF‐ or qEEG‐only models. Hence, the combination has potential for a more personalized prognosis in AD.